# TCRP1 transcriptionally regulated by c-Myc confers cancer chemoresistance in tongue and lung cancer

**DOI:** 10.1038/s41598-017-03763-0

**Published:** 2017-06-16

**Authors:** Xiaoting Jia, Zhijie Zhang, Kai Luo, Guopei Zheng, Minying Lu, Ying Song, Hao Liu, Huisi Qiu, Zhimin He

**Affiliations:** Affiliated Cancer Hospital & Institute of Guangzhou Medical University, Guangzhou, China

## Abstract

Previously, we cloned a new gene termed ‘tongue cancer resistance-associated protein 1’ (TCRP1), which modulates tumorigenesis, enhances cisplatin (cDDP) resistance in cancers, and may be a potential target for reversing drug resistance. However, the mechanisms for regulating TCRP1 expression remain unclear. Herein, we combined bioinformatics analysis with luciferase reporter assay and ChIP assay to determine that c-Myc could directly bind to TCRP1 promoter to upregulate its expression. TCRP1 upregulation in multidrug resistant tongue cancer cells (Tca8113/PYM) and cisplatin-resistant A549 lung cancer cells (A549/DDP) was accompanied by c-Myc upregulation, compared to respective parental cells. In tongue and lung cancer cells, siRNA-mediated knockdown of c-Myc led to decrease TCRP1 expression, whereas overexpression c-Myc did the opposite. Moreover, TCRP1 knockdown attenuated chemoresistance resulting from c-Myc overexpression, but TCRP1 overexpression impaired the effect of c-Myc knockdown on chemosensitivity. Additionally, in both human tongue and lung cancer tissues, c-Myc protein expression positively correlated with TCRP1 protein expression and these protein levels were associated with worse prognosis for patients. Combined, these findings suggest that c-Myc could transcriptionally regulate TCRP1 in cell lines and clinical samples and identified the c-Myc-TCRP1 axis as a negative biomarker of prognosis in tongue and lung cancers.

## Introduction

Cancer is becoming a serious public health problem in the world. In USA, it is evaluated that 1,685,210 cases of new cancer and 595,690 cancer-related deaths would be occurred in 2016^1^. In China, there were approximately 4,292,000 new cancer cases and 2,814,000 cancer deaths in 2015^2^. Tongue cancer and lung cancer are common subtypes, and both are with the characterized by rapid progression, fast metastasis, and poor prognosis^3, 4^. So far, surgical operation is the main treatment for cancer^5^, but chemotherapy was remarkably important in cancer therapy, and may be beneficial for controlling and narrowing local lesions before operation, preventing the recurrence and metastasis after operation, and may be the only viable therapy for inoperable late stage cancer. However, the chemoresistance becoming a major bottleneck to improve cancer treatment. Therefore, elucidating the mechanisms of chemoresistance is one of the key ways to identify targets for reversing drug resistance, and to improve the clinical treatment effects.

To explore the mechanisms of chemoresistance in cancer treatment, our research group previously established a multi-drug resistant cell line Tca8113/PYM derived from human tongue cancer line Tca8113, by stepwise selection using Pingyangmycin (PYM) as an inducing reagent^6^. PYM, also named bleomycin A5, was a famous drug for anti-tumor therapy. Utilizing microarray analysis of tongue cancer parent cell Tca8113 and its drug resistant cell Tca8113/PYM, Gu *et al*. identified a novel gene designated tongue cancer resistance-associated protein 1 (*TCRP1*) with a NCBI accession number of EF363480. *TCRP1* locating on chromosome 11q13.4, also known as FAM168A, was 1834 bp in length with open reading frame (ORF) 708 bp, encoded a protein of 235 amino acids^7^. The analysis of functional motifs showed that TCRP1 possessed one putative Crk Src homology 2 (SH2) binding domain and one Erk1 kinase motif ^8^. Immunofluorescence combined with western blot assay validated that TCRP1 located in nucleus and cytoplasm of A549 cells^9^. In our previous studies, we showed that TCRP1 inhibited apoptosis of oral squamous cell carcinoma (OSCC) cells with cDDP to improve its proliferation and survival ability^7^. It is validated that TCRP1 was positively correlated with poor prognosis and chemoresistance of OSCC patients^10^. Mechanically, the study suggested that TCRP1 could activate Akt and then upregulated NF-κB to enhance OSCC cells radioresistance^11^. Co-immunoprecipitation and western blot analysis suggested that TCRP1 interacted with two DNA repair proteins Pol β and INO80. Furtheremore, it is demonstrated that TCRP1 enhanced DNA repair via preventing Pol β degradation^8^. Recently, we found that TCRP1 mediated PI3K/Akt pathway activation to drive NIH/3T3 cells malignant transformation^12^. These previous studies suggest that targeting TCRP1 may be a potential method to reverse drug resistance. However, there is limited understanding of mechanisms for TCRP1 dysregulation in cancer cells.

Abnormal gene expression is related with changes in multi-stage regulation, such as the change in DNA or chromosome level, transcription, post-transcription, translation, and protein processing. Among these changes, transcriptional regulation as the first step in regulating gene expression is therefore the most important control point to determine gene expression. In eukaryotes, the transcriptional regulation contains cis-acting elements as promoters, trans-acting elements like transcriptional factors, epigenetic regulators such as histone acetylation, DNA methylation and so on. Promoters lie in 5′ UTR of gene transcript, where RNA polymerase II binds. Transcriptional factors, through binding to promoters and changing DNA conformation, regulate transcriptional activity of RNA polymerase II to ultimately regulate gene expression. Previous research has suggested that abnormal transcriptional regulation takes a important part in cancer development^13–15^. c-Myc, with a basic helix loop helix (bHLH) domain, usually binds to E-box sequences near the core promoter elements of target genes to enhance their expression^16^. Increasing research have found target genes of c-Myc in various tumor cells^17–19^. In the present study, we demonstrate that c-Myc transcriptionally upregulates TCRP1 in tongue and lung cancer cells through bioinformatics analysis combining with experimental validation. Moreover, we explored the expression relationships between c-Myc and TCRP1 in tongue and lung cancers, and found that c-Myc positively regulated TCRP1 protein expression, and the c-Myc-TCRP1 axis contributes to chemoresistance in tongue and lung cancers.

## Results

### c-Myc transcriptionally regulates TCRP1 in tongue and lung cancer cells

**Figure 1:**
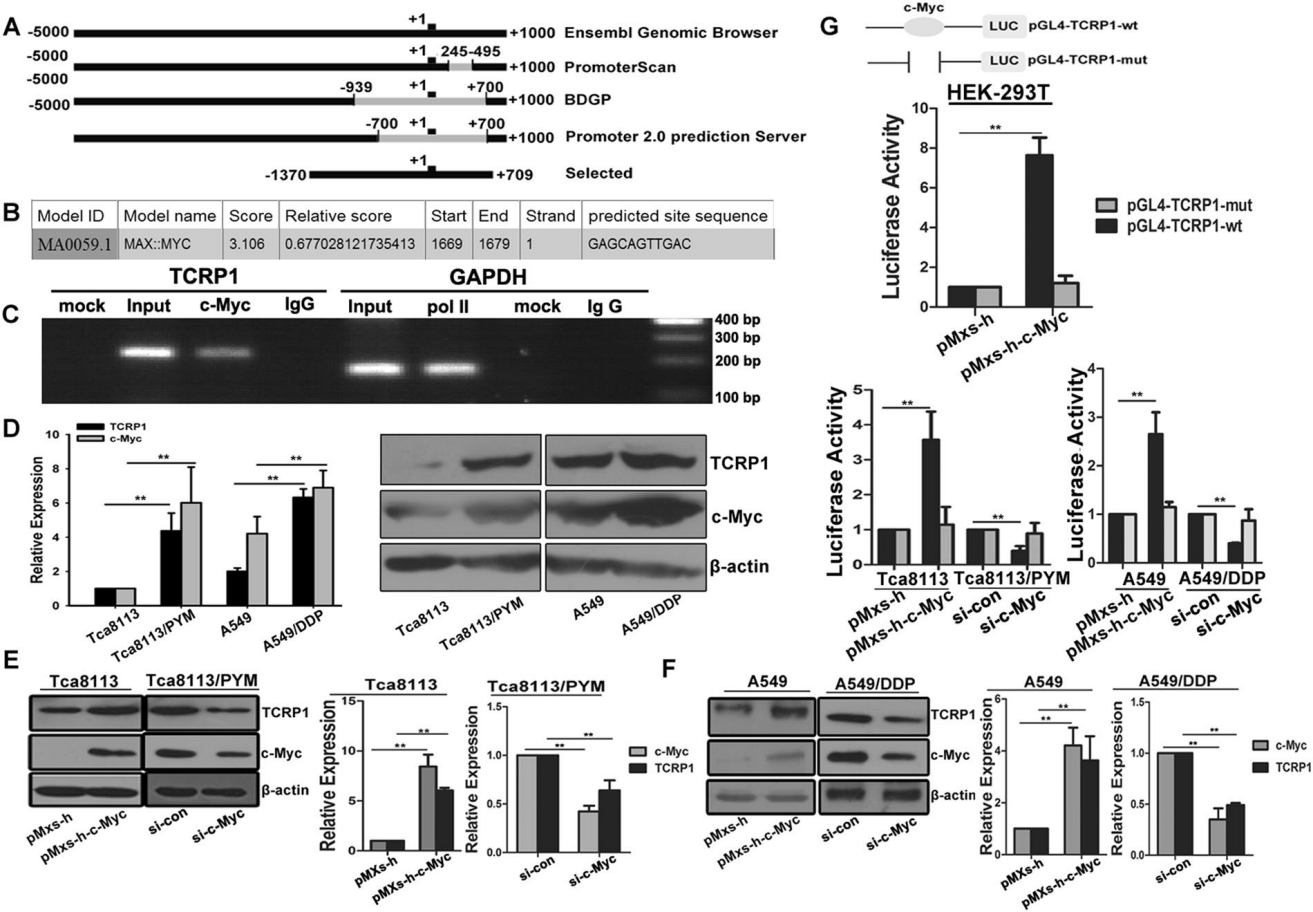
TCRP1 is upregulated by c-Myc in tongue and lung cancer cells. (**A**) A sketch map of potential TCRP1 promoter predicted by several online program. The transcriptional start site (TSS) of TCRP1 promoter was defined as‘+1’. (**B**) A schematic representation of c-Myc binding site in putative TCRP1 promoter. C-Myc also is known as Myc. (**C**) ChIP assay was performed in tongue cancer cell Tca8113/PYM. Pol IIcontacts to polymerase II. (**D**) TCRP1 and c-Myc expression were measured by real-time RT-PCR and western blot assay in tongue and lung cancer cells. **P < 0.01. (**E**,**F**) Plasmid pMxs-h-c-Myc and control pMxs-h were transfected into tongue cancer cell Tca8113 and lung cancer cell A549, siRNAs targeting c-Myc together with its control were transfected into Tca8113/PYM and A549/DDP cells, 48 h later, the mRNA and protein expression levels of TCRP1 and c-Myc were estimated in these cells. **P < 0.01. (**G**) A schematic diagram of c-Myc wild type and mutant binding site in TCRP1 promoter. Dual-luciferase reporter gene assay was conducted in HEK-293T, tongue and lung cancer cells with overexpression c-Myc or siRNA targeting c-Myc. **P < 0.01.

Through bioinformatics analysis, we selected 1370 bp before transcriptional start site (TSS) and 709 bp after TSS as the promoter of *TCRP1* (Fig. 1A). Then, the promoter sequence of *TCRP1* was submitted to the online program JASPAR (http://jaspar.genereg.net/) to identify possible transcriptional factors. There were potential binding sites for transcription factor c-Myc within *TCRP1* promoter (Fig. 1B). We performed ChIP assay to reveal that c-Myc directly bound to the potential *TCRP1* promoter in Tca8113/PYM cells (Fig. 1C). Moreover, we ascertained the levels of TCRP1 and c-Myc in tongue and lung cancer cells. The expressions of TCRP1 and c-Myc were much higher in chemoresistant cancer cells Tca8113/PYM and A549/DDP than those in parental cell line (Fig. 1D). As expected, ectopic overexpression of c-Myc *via* the pMxs-h-c-Myc construct upregulated TCRP1 both at mRNA and protein levels in Tca8113 and A549 cell lines. Conversely, TCRP1 mRNA and protein expression decreased following silencing c-Myc by siRNA in Tca8113/PYM and A549/DDP cells (Fig. 1E,F). To further investigate the effects of c-Myc on TCRP1 expression, we co-transfected c-Myc expression plasmid with wild type or mutant luciferase reporter plasmid into HEK-293T cells, and dual-luciferase reporter gene assay indicated that c-Myc remarkably increased luciferase activity of wild type but not mutant TCRP1 promoter (Fig. 1G). The similar results were obtained in Tca8113 and A549 cells. However, the wild type but not mutant TCRP1 promoter activity was significantly reduced by silencing c-Myc in Tca8113/PYM and A549/DDP cells (Fig. 1G). Combined, this data demonstrated that TCRP1 was transcriptionally regulated by c-Myc directly binding to its promoter.

### TCRP1 contributes to c-Myc-mediated chemoresistance in tongue and lung cancer cells

**Figure 2:**
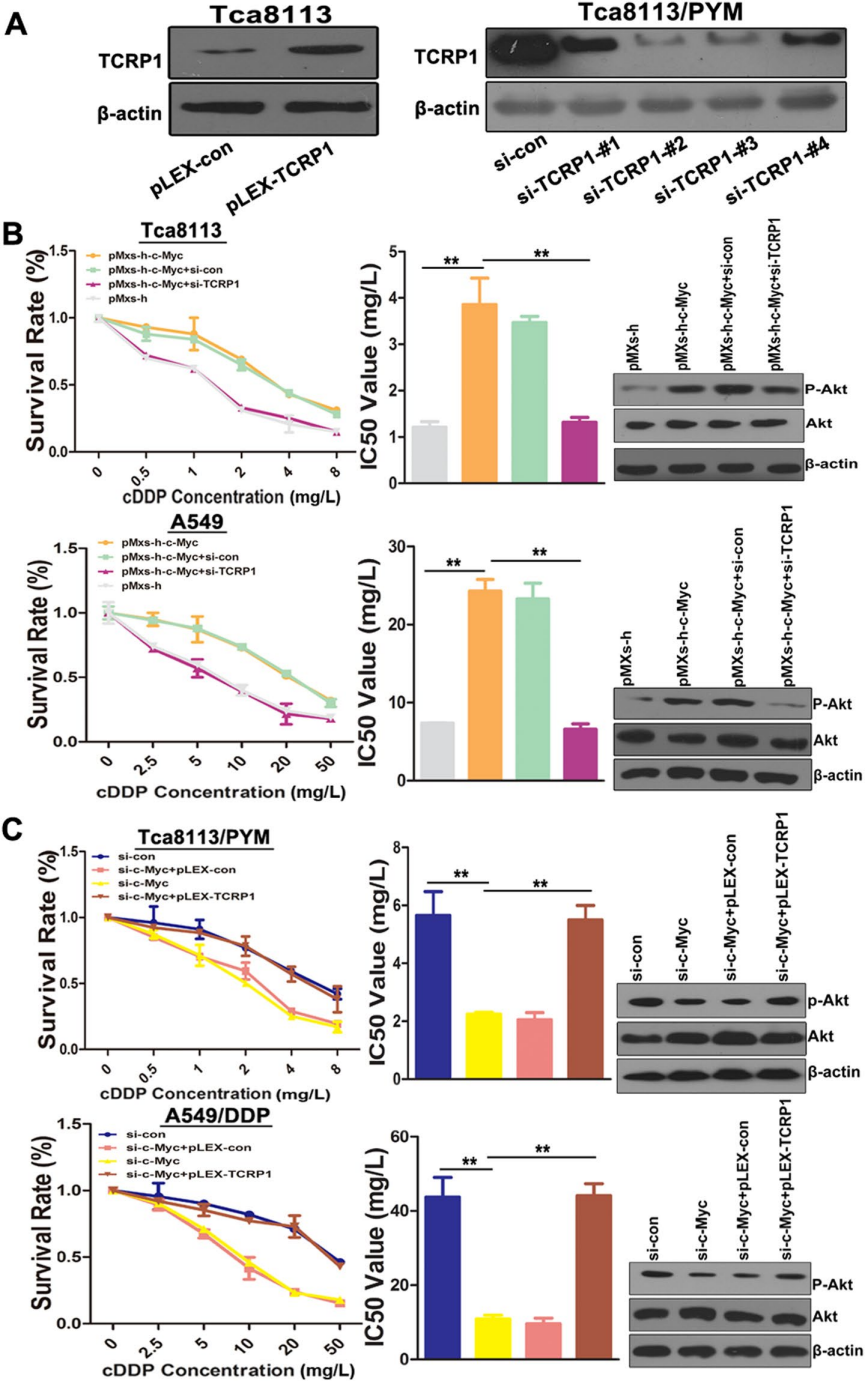
The c-Myc-TCRP1 axis inhibites sensitivity of tongue and lung cancer cells to cDDP. (**A**) Transfected plasmid pLEX-TCRP1 and its control into Tca8113 cells, meanwhile transfected siRNAs targeting TCRP1 and its control into Tca8113/PYM cells, 48 h later, western blot assay was used to measure TCRP1 expression levels in these different treated cells. (**B**) Overexpressed c-Myc together with silencing TCRP1 in parative cells Tca8113 and A549, (**C**) Silencing c-Myc together with over-expressed TCRP1 in chemoresistance cells Tca8113/PYM and A549/DDP, all these cells and each control group were treated with cDDP for 72 h, and the survival rate of these cells were detected by MTS assay, western blot assay was used to measure Akt activation. **P < 0.01.

The above data suggested that c-Myc was overexpressed in chemoresistance cells, and c-Myc positively regulated TCRP1 expression. Therefore, we investigated whether c-Myc modulated chemosensitivity of tongue and lung cancer cells *via* regulating TCRP1 expression. Initially, we transfected TCRP1 overexpression plasmid pLEX-TCRP1 and siRNAs targeting TCRP1 into Tca8113 and Tca8113/PYM cells, respectively. Western blot assays demonstrated that pLEX-TCRP1 plasmid could successfully overexpress TCRP1, and si-TCRP1-#2 was chosen as the most efficient siRNA oligonucleotide (Fig. 2A). Through MTS assay, we found that ectopic overexpression c-Myc in Tca8113 and A549 cells could remarkably increase the cDDP resistance and IC_50_ values of these cells (Fig. 2B). In contrast, knocking down c-Myc reduced the cDDP resistance of Tca8113/PYM and A549/DDP cells, and significantly decreased IC_50_ values of these treated cells (Fig. 2C). In addition, TCRP1 knockdown in Tca8113 and A459 cells evidently abolished their resistance to cDDP induced by c-Myc (Fig. 2B). Increasing TCRP1 reversed the effect of silencing c-Myc on chemotherapy of Tca/PYM and A549/DDP cells (Fig. 2C). Mechanically, western blot assay was performed to show that over c-Myc can upregulate phosphoylation of Akt, while knocking-down TCRP1 with siRNA can inhibited it in parental tongue and lung cancer cells (Fig. 2B). Similarly, overexprssion TCRP1 can enhance Akt activation prevented by silencing c-Myc in tongue and lung drug resistance cells (Fig. 2C). These results illustrated that TCRP1 was responsible for c-Myc-mediated chemoresistance in tongue and lung cancer cells.

### TCRP1 and c-Myc are positively correlated in tongue and lung cancer

**Figure 3:**
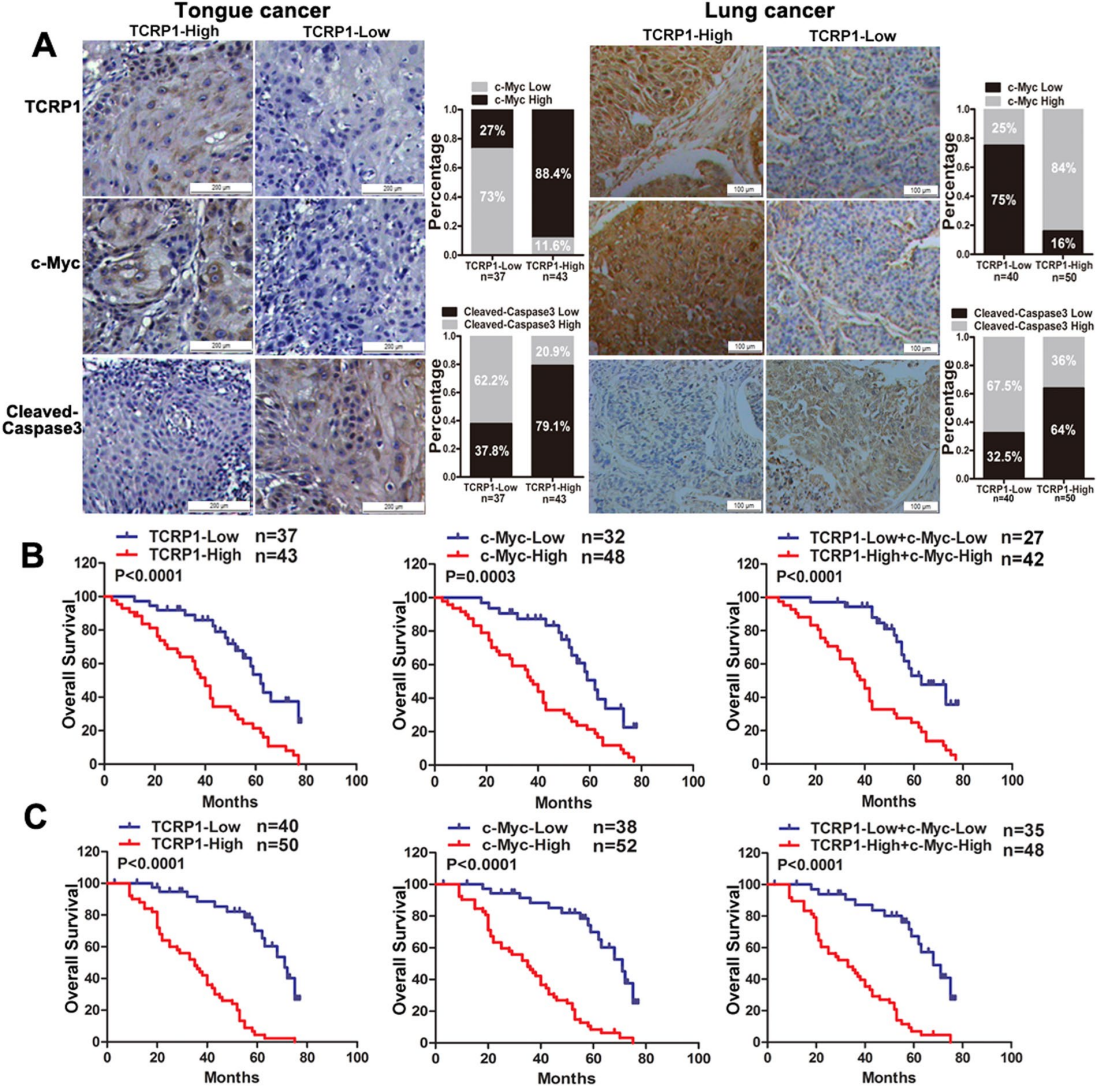
c-Myc positively related with TCRP1 and poor clinical outcome of patients. (**A**) Immunohistochemical assay was used to obtain c-Myc, TCRP1 and cleaved-caspase 3 expression in tongue and lung cancer tissues. Representative images of each protein expression was showed, and the relative expression pattern between TCRP1 and c-Myc or cleaved-caspase 3 were also analyzed. In tongue cancer tissues (**B**) and lung cancer tissues (**C**), Kaplan–Meier analysis was performed to estimate OS according to the protein level of TCRP1, c-Myc, even both them.

Since the data thus far had shown that c-Myc transcriptionally regulated TCRP1 expression and the c-Myc-TCRP1 axis mediated chemotherapy resistance in cancer cell lines, we next examined whether c-Myc was correlated with TCRP1 in tongue and lung cancer tissues. Immunohistochemical analysis was performed on 80 tongue cancer patients and 90 lung cancer patients having PYM and/or cDDP chemotherapy (Fig. 3A). The results showed that c-Myc was positively expressed in 38 of 43 cases tongue cancer tissues exhibiting high TCRP1 expression. Conversely, low expression of c-Myc was observed in 27 of 37 cases tongue cancer tissues exhibiting low TCRP1 protein expression (Fig. 3A). Similarly, the co-expression pattern of c-Myc and TCRP1 was also obtained in lung cancer. As shown in Fig. 3A, 75% lung cancer tissues with low TCRP1 protein level also expressed a low level of c-Myc, and 84% lung cancer tissues with high TCRP1 expression also showed a high level of c-Myc expression. Furthermore, we detected cleaved-caspase 3 expression levels in these tissues to show patients’ response to chemotherapy (Fig. 3A). The results showed that 62.2% tongue cancer tissues with lowly expressed TCRP1 increased cleaved-caspase 3 expression, exhibiting a more sensitivity to drug treatment. Meanwhile, 32 of 50 cases lung cancer tissues with TCRP1 high expression reduced cleaved-caspase 3 expression, and 27 of 40 cases lung cancer tissues with TCRP1 low expression enhanced cleaved-caspase 3 expression levels. This is strongly hinted that TCRP1 was negatively correlated with cleaved-caspase 3 and chemosensitivity of cancers. Kaplan–Meier analysis suggested that tongue cancer patients with high TCRP1 expression had a poorer prognosis than patients with low TCRP1 expression (Fig. 3B). We got the similar results in lung cancer patient samples (Fig. 3C). Moreover, c-Myc was negatively correlated with overall survival (OS) of tongue and lung cancer patients (Fig. 3B,C). In addition, the OS of patients with both high c-Myc and TCRP1 expression was much worse than that of patients with both low c-Myc and TCRP1 expression (Fig. 3B,C). In all, these results suggested a potential correlation expression pattern between c-Myc and TCRP1 in human tongue and lung cancer patients. c-Myc-TCRP1 axis could be a biomarker of poor prognosis of these patients.

## Discussion

Therapeutic resistance remains a major problem for cancer therapy^20^. However, mechanisms of therapeutic resistance are complex and are not completely understood in many cancers. Therefore, elucidating accurate molecular mechanisms of chemoresistance is of great importance in improving current treatments in the clinic. Previously, we identified that TCRP1 was related to chemoresistance in tongue cancer^7^ and was associated with lung cancer occurrence^12^; however, the mechanism of TCRP1 upregulation in cancer is still unclear. Here, we combined bioinformatics analysis with a series of experiments to ascertain that c-Myc directly bound to TCRP1 promoter and positively regulated its activity. We demonstrated in a MTS assay that c-Myc-mediated upregulation of TCRP1 enhanced chemoresistance of tongue and lung cancer cells. Finally, Kaplan–Meier analysis suggested that both c-Myc and TCRP1 were negative prognostic factors for tongue and lung cancers.

It is widely reported that c-Myc abnormally overexpressed in various cancers^21^. c-Myc can bind to the canonical element E-box (CACGTG) or non-canonical E-box (CANNTG) to transactivate targeted genes^22, 23^. A previous study showed that c-Myc induced ANRIL expression via directly binding to the E-box of its promoter^24^. Some reports revealed that c-Myc directly bound to DKC1 promoter and dramatically increased its expression^25^. Similarly, we found that there was a non-canonical E-box sequence (CAGTTG) on the TCRP1 promoter by bioinformatics analysis. Results of ChIP assay illustrated that c-Myc could directly bind to the E-box of TCRP1 promoter. Gain-of-function and loss-of-function assays suggested that c-Myc positively regulated TCRP1 expression in tongue and lung cancer cells. However, our results are in contrast to a previous report. That study revealed that more than with on-off effect on gene expression, c-Myc could amplify expression of the active genes, but not expression of immediate early genes, with a non-linear manner^26^.

Aberrant expression of c-Myc was implicated to role in chemoresistance^27^. It has been reported that c-Myc was enhanced in docetaxel-resistant lung adenocarcinoma (LAD) tissues comparing with docetaxel-sensitive tissues. Mechanically, c-Myc induced GSK-3β inactivation and subsequent Snail activation, contributed to EMT, and chemoresistance^28^. Several studies confirmed that carbon nanotubes (CNTs)-based drug delivery devices significantly enhanced cancer chemosensitivity^29^. It is reasoned that c-Myc, directly regulated the expression of ABC gene, was remarkably reduced in multiwalled carbon nanotubes (MWCNTs)-treated cells and ectopic overexpression c-Myc abolished MWCNTs’ inhibitory effects on ABC^29^. From solid transplantable tumor rat models treated with cDDP, researchers demonstrated that elevating c-Myc expression can sharply promote tumor cells treated with cDDP to survive^14^. A previous report also found that DYRK1A inhibited the proliferation and chemosensitivity of acute myeloid leukemia (AML) cell lines through downregulation c-Myc^30^. Peng *et al*. performed a series of experiments to validate that TCRP1 mediated cDDP resistance against DNA damage^10^. TCRP1, enhancing resistance to cDDP in Tca8113 cells through inhibited cDDP-mediated apoptosis in these cells^7^. Similarly, we also found that c-Myc contributed to chemoresistance in tongue and lung cancers. In this manuscript, we performed a series of experiments to determine whether c-Myc-TCRP1 mediates chemoresistance in tongue and lung cancer cells. We found that ectopic overexpression c-Myc enhanced the chemoresistance of tongue and lung cancer cells, while silencing TCRP1 abolished the effect of overexpressed c-Myc in these cells. On the contrary, knockdown of c-Myc sensitized tongue and lung cancer cells to cDDP, but overexpression TCRP1 reversed the effects induced by silencing c-Myc in these cells.

Moreover, c-Myc is a well-known poor prognosis marker in a variety of cancers. Studies have shown that patients with aberrant c-Myc or Bcl2 had significantly worse OS and progression-free survival (PFS), comparing with normal cases (P < 0.0001)^31^. In ALCL (anaplastic large cell lymphoma), Moritake *et al*. found that c-Myc overexpression was related to a high proliferation index and poor clinical outcome^32^. Moreover, it is noted that c-Myc and Ki-67 expression conferred poor OS on peripheral T-cell lymphomas patients, as indicated by the univariate analyses (p = 0.003 and 0.006, respectively). Additionally, it is showed that c-Myc acted as an independent prognostic factor of these patients (p = 0.004)^33^. In a similar way, our study showed that c-Myc and TCRP1 were positively correlated in tongue and lung cancer tissues. Kaplan–Meier analysis demonstrated that both c-Myc and TCRP1 were negative prognostic factors for OS in tongue and lung cancer patients. Cleaved-caspase 3 is a biomarker of cell apoptosis, and in current study we showed that cleaved-caspase 3 expression levels were enhanced in TCRP1 lowly expressed cancer tissues. It is confirmed that TCRP1 attenuated cell apoptosis. Future studies which focus on the molecular mechanism of c-Myc-TCRP1 axis responding to chemotherapy will be required. Moreover, we should develop new strategies to discover biochemical changes during chemotherapy.

## Material and Methods

### Cell culture and treatments

Tongue cancer cell line Tca8113 and lung cancer cell line A549 were cultured in RPMI-1640 (Gibco, Carlsbad, CA, USA) with 10% fetal bovine serum (FBS; Gibco, Carlsbad, CA, USA) at 37 °C, 5% CO_2_. Tca8113/PYM cells and A549/DDP cells were cultured in RPMI-1640 with 100 ng/ml PYM (Harbin Bolai Pharmaceutical, Harbin, China) or 1000 ng/ml cDDP (Sigma, USA), respectively. But these two cells should be maintained in PYM-free or cDDP-free medium for at least two weeks before experiments. HEK-293T cells were cultured in DMEM (Gibco, Carlsbad, CA, USA) containing 10% FBS at 37 °C, 5% CO_2_.

### Bioinformatics analysis for identifying TCRP1 promoter and transcriptional factors

Through Ensemble Genome Browser website (http://asia.ensembl.org/index.html), we first downloaded TCRP1 sequence, labeled the transcriptional start site (TSS) of TCRP1 was +1, extracted upstream 5000 bp sequence and downstream 999 bp sequence of TSS, and then submitted this 6000 bp sequence onto programs to identify the promoter region. The online programs we used were PromoterScan (http://www-bimas.cit.nih.gov/molbio/proscan), BDGP (http://www.fruitfly.org/seq_tools/promoter.html), and Promoter 2.0 Prediction Server (http://www.cbs.dtu.dk/services/Promoter/). Finally, we submitted the promoter sequence of TCRP1 to identify possible transcriptional factors *via* the online program JASPAR (http://jaspar.genereg.net/).

### RNA extraction and real-time RT-PCR assays

We extracted total RNAs using TRIzol and synthesized them into cDNA *via* the first-strand synthesis system (Thermo Scientific, USA). Real-time RT-PCR was performed following the standard protocol on ABI 7500fast with Maxima SYBR Green/ROX qPCR Master Mix (2X) (Thermo scientific, USA). We choosed *GAPDH* as an internal control and listed all primers used in Table 1.

**Table 1:** Primers list in this study.

Primers	Sequence (5′-3′)
**For ectopic overexpression** ^**a**^	
pMXs-h-c-myc-F	CGG**GGATCC**ATGCCCCTCAACGTTAGCTTC
pMXs-h-c-myc-R	CGG**CTCGAG**ACGCACAAGAGTTCCGTAGCTGT
pLEX-TCRP1-F	CGG**GGATCC**ATGAACCCTGTTTACAGCCCCC
pLEX-TCRP1-R	CGG**CTCGAG**ACGTGCCCCCACACTGGTAA
**For dual-luciferase reporter assay** ^**a,b**^	
pGL4-TCRP1-wt -F	CCG**CTCGAG**GGGACTGAATGAGGGTGGTGAT
pGL4-TCRP1-wt-R	CCC**AAGCTT**CTCCGTTGAAATCATTCCCCTG
pGL4-TCRP1-mut-F	tcatctACAGCTTGGGATCTGAGC
pGL4-TCRP1-mut-R	CTCCAGGCCAAACCCAAATC
**For ChIP**	
ChIP-c-Myc -F	AGGAGGACTTTTGATGGAGAATAACC
ChIP-c-Myc -R	CTCCGTTGAAATCATTCCCCTG
ChIP-GAPDH-F	**TACTAGCGGTTTTACGGGCG**
ChIP-GAPDH- R	TCGAACAGGAGGAGCAGAGAGCGA
**For real-time RT-PCR**	
TCRP1-F	CCAATAGTCCCAGTTATGCTCCA
TCRP1-R	TGCTTGGTAAGTTCGGTTCTCG
c-Myc-F	CACCGAGTCGTAGTCGAGGT
c-Myc-R	TTTCGGGTAGTGGAAAACCA
GAPDH-F	ATTCCATGGCACCGTCAAGGCTGA
GAPDH-R	TTCTCCATGGTGGTGAAGACGCCA

^a^Nucleotides under “bold” indicate restriction sites introduced for cloning.

^b^Nucleotides in lower-case are the mutant sites.

### Western Blot Assay

Cells were seeded overnight, and we transfected plasmids into cells via Lipofectamine 2000 reagent (Life Technologies, USA). 48 h post-transfected, cells were incubated in mammalian cell lysis reagent (Thermo Scientific, USA) with protease inhibitor cocktail (Roche, Switzerland) for 20 mins at 4 °C, centrifuged at 13000 g for 15 mins, the supernatant was harvested as the total protein. Next, 70 µg total protein per lane was loaded on 10% SDS-PAGE gel. And protein bands were transferred from gel to PVDF membranes at 70 V pressure for 1 h, blocked overnight with 5% non-fat milk at 4 °C, followed by sequential incubation with primary antibodies and secondary antibodies. Antibodies used were as follows: TCRP1 antibody (Santa Cruz Biotechnology, 1:300), c-Myc antibody (Cell Signaling Technology, 1:4000), β-actin antibody (Cell Signaling Technology, 1:4000), and anti-rabbit secondary antibody (Merck, 1:10000). Finally, we used chemiluminescent detection system (Millipore, USA) to detect signal.

### Construction plasmids

Total RNA was isolated from Tca8113 cells and was inversely transcripted into cDNA, c-Myc ORF (open reading frame) and TCRP1 ORF were amplified by PrimeSTAR^®^ HS DNA Polymerase (TaKaRa, Japan), and primers are showed in Table 1. The c-Myc fragment and pMxs-h vector (Promega, USA), and TCRP1 fragment and pLEX-MCS vector (Thermo Scientific, USA) were digested by restriction enzyme BamH *I* (Thermo scientific, USA) and Xho *I* (Thermo scientific, USA). After digestion, the c-Myc fragment were ligased into pMxs-h vector and TCRP1 fragment were ligased into pLEX-MCS vector by T4 DNA ligase (Thermo scientific, USA), and then sequenced (Life Technologies, USA), named pMxs-h-c-Myc and pLEX-TCRP1, respectively. We mutated c-Myc binding site “CAGTTG” to “TCATCT” within TCRP1 promoter following instructions of MutanBEST Kit (TaKaRa, China), and respectively cloned wild type (wt) and mutant TCRP1 promoter into a luciferase reporter vector pGL4, named pGL4-TCRP1-wt and pGL4-TCRP1-mut. Plasmids with the expected mutations were sequenced to confirm the presence of the mutation.

### Transfection and dual-luciferase reporter assays

For western blot and real-time RT-PCR assay, cells were seeded overnight in 6-well plates, 4 μg plasmids were transfected per well as described by the manufacturer. After 48 h transfection, mRNA and protein were extracted from these cells, and then real-time RT-PCR and western blot assay were performed.

For dual-luciferase reporter assays, cells were seeded overnight in 96-well plates, and then 0.3 μg c-Myc overexpression plasmid, 0.2 μg TCRP1 promoter-luciferase reporter plasmid, and 0.02 μg pRL-TK plasmid (Promega, USA) were co-transfected per well *via* Lipofectamine 2000. The internal control we choosed was Renilla luciferase plasmid pRL-TK. After 48 h, dual-luciferase reporter assay system (Promega, USA) was used to evaluate both firefly and Renilla luciferase activity.

### Chromatin immunoprecipitation assay (ChIP)

We used EZ-CHIP^TM^ chromatin immunoprecipitation kit (Millipore, Germany) to perform ChIP assay. Tca8113/PYM cells were crosslinked by 1% formaldehyde for 10 mins at room temperature. Glycine was added to stop this reaction for 5 mins, and then cells were pelleted and incubated on ice for 30 mins with 1 × Lysis Buffer containning 1 × Protease Inhibitor Cocktail II. Cells were sheared on ice by sonication (40 pulses, 20 sec/pulse at 30% power) and centrifugation. Protein G agarose was added into the supernatant for 1 h at 4 °C, and then the supernatent was incubated with c-Myc antibody and Polymerase II antibody at 4 °C overnight, respectively. Proteinase K was used to digest proteins, the chromatin was extracted and used in PCR analysis. Primers were listed in Table 1. PCR products were run on 3% agarose gels to visualize the presence of TCRP1 promoter.

### Tissue microarray (TMA) and Immunohistochemical analysis

We collected 80 cases of tongue cancer and 90 cases of non small cell lung cancer (NSCLC) for immunohistochemical analysis. These tissues were collected from patients undergoing surgery at Affiliated Cancer Center Hospital & Institute of Guangzhou Medical University. Each patients have accepted chemotherapy before surgery. All procedures performed in studies involving human participants were in accordance with Declaration of Helsinki principles and the guidelines of the International Committee of Medical Journal Editors. This project was agreed by Affiliated Cancer Center Hospital & Institute of Guangzhou Medical University IRB. All individual patients provided written informed consent for biological studies at their initial presentation. Biopsy specimens were assembled and provided to Shanghai Outdo Biotech Company for preparing TMA. Core size was 0.6 cm, and each represented a different tumor sample.

The TMA sections were dewaxed and rehydrated, and then Hydrogen Peroxide Block was used to cover the sections for 10 mins. Protein Block was applied for 10 minutes at room temperature to block nonspecific background staining. The c-Myc antibody (abcam) was diluted at 1:200, TCRP1 antibody (FAM168A antibody, sigma) was used at a dilution of 1:150, and cleaved-caspase 3 antibody (Cell signaling Technology) was diluted at 1:300. Immunostaining was performed with Rabbit/Mouse Specific HRP/DAB (ABC) Detection IHC Kit (abcam) following manufacturer’s instructions. Two independent pathologists were asked to analyze the same slide. We considered the extent and intensity of immunoreactivity when evaluating protein expression levels in these tissues. The extent of staining was scored from 0% to 100%, and intensity of staining was from 0 to 4. The final results was obtained by multiplying the 2 scores.

### Statistical analysis

All experiments were carried out for more than three times, data were showed as mean ± standard error. Statistical package for the Social Sciences Version 19.0 (SPSS 19.0) was used to analyze data and statistical analysis was done by Student’s *t* test. Comparisons between groups in Immunohistochemical analysis were done by χ^2^ test. *P* < 0.05 was considered statistically significant.

## References

[1] Siegel RL, Miller KD, Jemal A (2016). Cancer statistics, 2016. CA Cancer J Clin..

[2] Chen W (2016). Cancer statistics in China, 2015. CA Cancer J Clin..

[3] Kim SH (2006). Correlations of oral tongue cancer invasion with matrix metalloproteinases (MMPs) and vascular endothelial growth factor (VEGF) expression. J Surg Oncol..

[4] Bremnes RM, Veve R, Hirsch FR, Franklin WA (2002). The E-cadherin cell-cell adhesion complex and lung cancer invasion, metastasis, and prognosis. Lung Cancer..

[5] O’Rourke N, Edwards R (2000). Lung cancer treatment waiting times and tumour growth. Clin Oncol..

[6] Zheng G (2010). Identification of carbonic anhydrase 9 as a contributor to pingyangmycin-induced drug resistance in human tongue cancer cells. FEBS J..

[7] Gu Y (2011). Cloning and functional characterization of TCRP1, a novel gene mediating resistance to cisplatin in an oral squamous cell carcinoma cell line. FEBS Lett..

[8] Liu X (2015). TCRP1 contributes to cisplatin resistance by preventing Pol beta degradation in lung cancer cells. Mol Cell Biochem..

[9] Liu, X., Gu, Y., Wang, C., Zheng, G. & He, Z. TCRP1-mediated DNA damage repair is involved in the radioresistance in lung cancer cells. *Oncol Lett*. (accept) (2016).

[10] Peng B, Yi S, Gu Y, Zheng G, He Z (2012). Purification and biochemical characterization of a novel protein-tongue cancer chemotherapy resistance-associated protein1 (TCRP1). Protein Expr Purif..

[11] Gu Y (2011). TCRP1 promotes radioresistance of oral squamous cell carcinoma cells via Akt signal pathway. Mol Cell Biochem..

[12] Wang, C., *et al*. TCRP1 promotes NIH/3T3 Cell Transformation by over-activating PDK1 and AKT1. *Oncogenesis*. (accept) (2017). 10.1038/oncsis.2017.18 PMC5520495 28436990

[13] Li Q (2009). Critical Role and Regulation of Transcription Factor FoxM1 in Human Gastric Cancer Angiogenesis and Progression. Cancer Res..

[14] Walker TL, White JD, Esdale WJ, Burton MA, DeCruz EE (1996). Tumour cells surviving *in vivo* cisplatin chemotherapy display elevated c-myc expression. Br J Cancer..

[15] Yamane K (2007). PLU-1 Is an H3K4 Demethylase Involved in Transcriptional Repression and Breast Cancer Cell Proliferation. Mol Cell..

[16] Lin CY (2012). Transcriptional amplification in tumor cells with elevated c-Myc. Cell..

[17] Dang CV (2006). The c-Myc target gene network. Semin Cancer Biol..

[18] Kim YH (2006). Combined microarray analysis of small cell lung cancer reveals altered apoptotic balance and distinct expression signatures of MYC family gene amplification. Oncogene..

[19] Sabò A (2014). Selective transcriptional regulation by Myc in cellular growth control and lymphomagenesis. Nature..

[20] Wang W (2016). Effector T Cells Abrogate Stroma-Mediated Chemoresistance in Ovarian Cancer. Cell..

[21] Chen BJ, Wu YL, Tanaka Y, Zhang W (2014). Small molecules targeting c-Myc oncogene: promising anti-cancer therapeutics. Int J Biol Sci..

[22] Guccione E (2006). Myc-binding-site recognition in the human genome is determined by chromatin context. Nat Cell Biol..

[23] Solomon DL, Amati B, Land H (1993). Distinct DNA binding preferences for the c-Myc/Max and Max/Max dimers. Nucleic Acids Res..

[24] Lu Y (2016). Long noncoding RNA ANRIL could be transactivated by c-Myc and promote tumor progression of non-small-cell lung cancer. Onco Targets Ther..

[25] O’Brien R (2016). MYC-Driven Neuroblastomas Are Addicted to a Telomerase-Independent Function of Dyskerin. Cancer Res..

[26] Nie Z (2012). c-Myc is a universal amplifier of expressed genes in lymphocytes and embryonic stem cells. Cell..

[27] Klapproth K, Wirth T (2010). Advances in the understanding of MYC-induced lymphomagenesis. Br J Haematol..

[28] Chen D (2014). MicroRNA-451 induces epithelial-mesenchymal transition in docetaxel-resistant lung adenocarcinoma cells by targeting proto-oncogene c-Myc. Eur J Cancer..

[29] Wang Z (2015). Suppression of c-Myc is involved in multi-walled carbon nanotubes’ down-regulation of ATP-binding cassette transporters in human colon adenocarcinoma cells. Toxicol Appl Pharmacol..

[30] Liu Q (2014). Tumor suppressor DYRK1A effects on proliferation and chemoresistance of AML cells by downregulating c-Myc. PLoS One..

[31] Lu TX (2015). MYC or BCL2 copy number aberration is a strong predictor of outcome in patients with diffuse large B-cell lymphoma. Oncotarget..

[32] Moritake H (2011). C-MYC rearrangement may induce an aggressive phenotype in anaplastic lymphoma kinase positive anaplastic large cell lymphoma: Identification of a novel fusion gene ALO17/C-MYC. Am J Hematol..

[33] Manso R (2016). C-MYC is related to GATA3 expression and associated with poor prognosis in nodal peripheral T-cell lymphomas. Haematologica..

